# The Potential of *Helichsryum splendidum* (Thunb.) Less. for the Restoration of Sites Polluted with Coal Fly Ash

**DOI:** 10.3390/plants13182551

**Published:** 2024-09-11

**Authors:** Alexis Munyengabe, Ledwaba Samuel Kamogelo, Titus Yeliku-ang Ngmenzuma, Maria Fezile Banda

**Affiliations:** 1 Department of Chemistry, Faculty of Science, Arcadia Campus, Tshwane University of Technology, Pretoria 0183, South Africa; munyengabea@tut.ac.za (A.M.); samledwaba07@gmail.com (L.S.K.); 2 Department of Crop Sciences, Faculty of Science, Arcadia Campus, Tshwane University of Technology, Pretoria 0183, South Africa; zuma.titus@yahoo.com

**Keywords:** phytoremediation, *Helichrysum splendidum* (Thunb.) Less., translocation factor, bioconcentration factor, trace metals, gas-exchange analysis

## Abstract

The disposal of coal fly ash (CFA) generated from coal-fired power stations has serious impact on the ecosystem, by converting large pieces of land to barren ash dams with the potential to contaminate groundwater, surface water, air and soil. The aim of this study was to clarify the potential of phytoremediation using *Helichrysum splendidum* (Thunb.) Less. in areas polluted by CFA through conduction of pot trial experiments for 14 weeks. Plants of the same age were cultivated in CFA to assess their growth, photosynthetic rate and tolerance towards metal toxicity. This study revealed that the CFA was moderately polluted with heavy metals, and a lower photosynthetic rate was recorded for the CFA plants in comparison to the controls (plants grown in soil). Although the CO_2_ assimilation rate was lower for the CFA plants, increased growth was recorded for all the plants tested. Inductively coupled plasma mass spectrometry (ICP-MS) was used to quantify the amount of trace elements in samples and parameters including translocation factor (TF) and bioconcentration factor (BCF) were used to evaluate the phytoremediation potential of *H. splendidum* (Thunb.) Less. The results revealed that higher concentrations of Cd, Co, Cr, Cu, Mn and Pb were accumulated in the roots, while As, Ni and Zn were found in the shoots. Elements including As, Cr and Zn reported TF values above 1, indicating the plants’ phytoextraction potential. The BCF values for As, Cu and Zn were 1.22, 1.19 and 1.03, indicating effectiveness in the phytostabilization processes. A removal rate efficiency ranging from 18.0 to 56.7% was recorded confirming that, *H. splendidum* (Thunb.) Less. can be employed for restoration of CFA dams.

## 1. Introduction

Thermoelectric power plants that burn the coal to generate electricity produce solid by-products including boiler slag, bottom ash, flue gas desulphurization material and coal fly ash (CFA) [1]. Amongst them, CFA is the one generated in large quantities and is disposed of in ash dams constructed in the vicinity of the power stations. The dry and wet methods are the most well-known disposal techniques of CFA, which are not effective and highly expensive to maintain. Due to much reliance in coal for power generation, the production of CFA is anticipated to reach more than 1 billion tons by 2030, as its utilization rate does not counterbalance its production rate in some countries [2]. The utilization rate differs from country to country, with highest reported in Denmark (100%), Italy (100%), the Netherlands (100%), Japan (96.4%), France, Australia and Germany (85%), Canada (75%), and the USA (65%) [3,4,5]. The annual production of CFA in South Africa is around 26 million tons, of which less than 10% is recycled, and the remaining is stored in disposal sites, converting large pieces of land to unusable sites [6]. The disposal of CFA is an increasing economic and environmental challenge, and therefore, it is imperative that other sustainable methods of managing CFA can be developed [7].

CFA is largely characterized by metal oxides including SiO_2_, Al_2_O_3_, CaO, Fe_2_O_3_, MgO, Na_2_O and K_2_O, and these greatly influence its pH. The pH values of CFA can be categorized into three ranges: slightly alkaline (pH 6.5–7.5), moderately alkaline (pH 7.5–8.5) and highly alkaline (pH > 8) [8,9]. The high concentration of soluble salts and the pH values are unfavorable conditions for plant growth, and as such, CFA disposal sites are barren lands with no vegetation. Although some nutrients are available in CFA, it lacks phosphorus and nitrogen, which are highly needed for plant growth. Furthermore, the toxicity of CFA has led to a reduced number of the microorganisms required to fix nitrogen and improve the fertility of the substrate [10,11]. Many reports have highlighted the presence of organic and inorganic pollutants in CFA. Trace metals, as well as persistent organic pollutants such as polychlorinated biphenyls (PCBs) and polycyclic aromatic hydrocarbons (PAHs), have been reported in CFA, indicating its contribution to environmental pollution [12].

The small particles of CFA are easily dispersed into the environment during windy seasons and as such can travel far distances, resulting in air pollution. Toxic elements can leach into groundwaters and pollute the water, resulting in soil, surface and groundwater pollution [13]. There are some conventional remediation methods to clean up contaminated sites, specifically land contaminated with trace metals. In spite of being efficient, these methods are time consuming, environmentally devastating and expensive [14]. Phytoremediation techniques are cost-effective and have long-term applicability and aesthetic advantages, hence they have been approved as suitable methods of cleaning up polluted areas and reducing pollutants in the soil and other contaminated sites [15,16]. Referring to Zgorelec et al. [17], phytoremediation techniques do not upset or damage the surrounding environment, nor do they require any additional energy input. They can provide long-term plant cover, which may reduce surface runoff and erosion, as well as minimize the availability and mobility of trace metals in the environment, therefore preventing their transfer to subsequent links of the food web and the migration of toxic ions into groundwaters [10,17,18].

Phytostabilization and phytoextraction are the most used techniques in phytoremediation, and the difference in their mechanisms is that in a phytostabilization method the plant is able to immobilize the pollutants in the root system, while in a phytoextraction process pollutants are translocated into the shoots of the plant [17,19,20,21]. *Zea mays* L. was confirmed as a good phytostabilizer for soils contaminated with Zn and Cd [22]. Plants with a rapid growth rate, high biomass and high tolerance to toxic metals are ideal candidates for phytoextraction, as they can transfer metals to the harvestable plant parts [23,24].

The phytoremediation potential of plants species can be evaluated by determining their translocation factor (TF) and bioconcentration factor (BCF) [25]. The BCF is an index of the capacity of the plant to accumulate metals into its roots with respect to its concentration in environmental matrix, as shown in Equation (1) (Section 2.6) [11,14,19,26]. A plant with a BCF value greater than 1 is considered as a good candidate for phytostabilization [26,27]. The TF index indicates the ability of the plant to translocate metals from the roots to its aerial parts, as shown in Equation (2) (Section 2.6), and values above 1 indicate potential for phytoextraction [10,14,16,28]. The practical application of the phytoremediation process considers the use of plant species that do not require high maintenance and can grow and survive in harsh conditions for longer periods. Despite the potential of the *Helichrysum* genus for phytoremediation processes, only a few plants from this family have received attention [16,29,30].

Species including *Helichrysum italicum*, *Helichrysum candolleanum*, *Helichrysum decumbens* and *Helichrysum splendidum* (Thunb.) Less. have demonstrated potential for phytoremediation of contaminated soils, but no work has been reported for restoration of CFA deposits using *H. splendidum* (Thunb.) Less. *H. splendidum* (Thunb.) Less. is a perennial plant that can grow effortlessly in adverse conditions such as hot weather conditions, wetlands and in soils polluted with toxic metals and therefore is an ideal candidate to remediate South African sites polluted with CFA. The aim of this paper is to clarify the potential of phytoremediation using *H. splenididum* (Thunb.) Less.

## 2. Methodology

### 2.1. Sampling Site Description and Physicochemical Parameters of CFA and Soil

A CFA sample was collected from the Eskom, Hendrina power station located in Mpumalanga Province (26°2′ S 29°36′ E). Soil samples were collected some distance away from the ash dams around the power station and were used as controls. Portions of the samples were air-dried, homogenized and stored in glass bottles until further analysis. The pH and electrical conductivity (EC) of the samples were determined by weighing 5 g of CFA or soil, and transferred transferring to 50 mL centrifuge vials and added 10 mL of accurately measured deionized water to each sample. The mixtures were placed on a Labcon platform shaker (Laboratory Marketing Services CC, Maraisburg, South Africa) at 200 rpm for 12 h and then centrifuged for 10 min at 5000 rpm. A pH/conductivity combimeter (Orion Star Series Meter Thermo Fischer Scientific Inc., Beverly, MA, USA) was used to measure the pH and EC of the samples. The Walkley and Black method (chromic acid titration) was used to determine the total organic carbon (TOC) content of the samples, where 0.16 M of a potassium dichromate (K_2_Cr_2_O_7_) standard, 0.50 M ferrous sulphate heptahydrate (FeSO_4_.7H_2_O) solution and 0.025 M of an ortho-phenanthroline indicator were prepared [31]. An amount of 0.50 g of the soil or CFA was weighed for each sample and transferred to a 250 mL conical flask. An amount of 10 mL of the K_2_Cr_2_O_7_ was added to each sample, followed by the addition of 20 mL of concentrated sulfuric acid, and the mixture was left to stand for 30 min in the fume hood. Then, 200 mL of deionized water was added to the mixture, followed by 10 drops of ortho-phenanthroline indicator, and the solution was titrated using the FeSO_4_.7H_2_O solution.

### 2.2. Pot Trials

*Helichrysum splendidum* (Thunb.) Less. plants of the same age (twelve weeks), purchased from Random Harvest Nursery (Pretoria, South Africa), were used for the pot trials. The experiment was conducted in an open space under natural conditions during the period from March to June 2023. Plants were transplanted into 4-L plastic pots, with a 20 cm bottom diameter, and were filled to a depth 18 cm with CFA or soil. The experiment included 15 CFA-grown plants and 3 untreated plants growing in the collected soils were used as controls. The plants were watered thrice a week using tap water and were monitored for 14 weeks. Using a measuring tape, the lengths of the longest stems, from the root–stem junction to the leaf apex, were measured every two to three weeks for a period of fourteen weeks to evaluate plant growth.

### 2.3. Gas-Exchange Measurements

Gas-exchange measurements were used to evaluate the physiological properties of the plants, including CO_2_ assimilation or photosynthetic rate (A), transpiration efficiency (E), stomatal conductance to water vapor (Gs) and intercellular CO_2_ (Ci) on a leaf.

Photosynthetic measurements were taken on fully expanded young trifoliate leaves at the same position from the apex for triplicate plants per treatment (*n* = 3) and between 08:00 and 11:00 am at days 25 and 40 after planting using a portable infra-red gas analyzer photosynthesis system (Li-6400XT, Li-COR instrument, Nebraska, Lincoln, OR, USA) as described by Makoi et al. [32]. Leaves were allowed to acclimate to the light environment in the chamber for 4 to 5 min before each measurement was taken. The instrument was calibrated to the following conditions in the leaf chamber before use: light intensity 1000 μmol photons m^−2^ s^−1^, reference CO_2_ concentration 400 ppm, flow rate 400 μmol s^−1^, leaf temperature 25 °C and a relative humidity of 44%.

### 2.4. Plant Harvest

Following the fourteen-week period during which the pot trials were conducted, each entire plant was uprooted from the pot and thoroughly cleaned up with tap water to eliminate any remaining soil or CFA, then rinsed first with 2% HNO_3_ and finally with distilled water. Plants were separated into roots and shoots and allowed to air dry. Once dried, the plants were milled using a ball miller (BM500, Anton Paar, Midrand, South Africa) at 15 Hz for 15 min, sieved (<2 mm) to a homogenous sample and stored at room temperature until further use. Soil and CFA samples from the pots, after harvest, were also collected in plastic centrifuge vials and allowed to air dry for further analysis.

### 2.5. Metal Content Analysis

Acid digestion was performed on control soil and CFA samples (before and after the pot trial experiment), a certified reference material (CRM) and plant parts in order to conduct a full elemental analysis using inductively coupled plasma mass spectrometry (ICP-MS). Method validation was conducted using the CRM (SRM 1944, New York Waterway Sediments, NIST, New York, USA). The shoots of *H. splendidum* (0.25 g) were accurately weighed and transferred into a conical flask, and 5 mL of 65% HNO_3_ *w*/*w* was measured and added into each sample. The mixtures were placed on a hot plate at 110 °C for 30 min, and the resulting digests were diluted to 25 mL using deionized water. For the soil, CFA and CRM samples, a combination of 65% HNO_3_ and 32% HCl was used. First, we accurately weighed the samples (0.25 g), then added 20 mL HCl:HNO_3_ (5:15) and heated for 30 min and then added 2 mL hydrogen peroxide (*w*/*w*) and further heated for extra 15–30 min. The resulting digests were also diluted to 25 mL using deionized water. The mixtures were then filtered using micro-filters and stored in centrifuge vials until further analysis. The samples were further diluted to a total volume of 10 mL by combining 100 µL of sample and 9.9 mL of deionized water.

ICP-MS was used for the analysis and determination of metal concentrations in the CFA and control soil before and after the pot trial experiment as well as in the CRM and plant materials. The calibration of the instrument was conducted using standards prepared from a 1000 mg L^−1^ multi-elemental standard stock solution (Fluka A.G., Buchs, Switzerland). The working standard solutions with concentrations of 200, 400, 600, 800 and 1000 µg L^−1^ were prepared by dilution of the stock. The instrument’s operating parameters were set as follows: nebulizer flow rate (0.88 L min^−1^), ICP RF power (1500 W), auxiliary gas flow rate (1.2 L min^−1^), plasma gas flow (18 L min^−1^) and sample uptake rate (1.6 mL min^−1^).

### 2.6. Phytoextraction and Phytostabilization Potential of the Plant

Two indices were used to assess the phytostabilization and phytoextraction potential of trace metals in the plant parts, Bioconcentration Factor (BCF) and Translocation Factor (TF), using Equations (1) and (2) [25,33].

(1) $$BCF=\frac{Concentration\;of\;a\;metal\;in\;plant\;root}{Concentration\;of\;a\;metal\;in\;CFA\;or\;soil}$$

(2) $$TF=\frac{Concentration\;of\;a\;metal\;in\;plant\;shoot}{Concentration\;of\;a\;metal\;in\;plant\;root}$$

### 2.7. Statistical Analysis

The data were subjected to analysis of variance (ANOVA) to compare means of the treatments using the STATISTICA program (version 10.1). Where there were differences, the Duncan’s multiple range test was used to separate the means at *p* ≤ 0.05.

## 3. Results and Discussion

### 3.1. CFA and Control Soil Characterizations before and after Experiment

The physical and chemical parameters of control soil and CFA collected from Hendrina power station, before and after the pot trials, are presented in Table 1. The parameters, including EC, pH and TOC, were measured, and they were significant in both CFA and control soil, as indicated by their F-statistic values. The pH of control soil increased from 5.77 to 6.51, while for CFA pH decreased after 14 weeks of treatment. The pH of CFA decreased from 7.92 to 7.47, which indicates an improvement of properties, as plants thrive in a pH that is slightly acidic to neutral. The reduced pH may be attributed to the organic acids produced by root exudates releasing the H^+^ ion and therefore improving the CFA’s physicochemical properties [34]. The recorded values suggest improved properties, which better supports microbial activity and results in increased nutrient availability.

The EC values of all the samples were increased, with control soils increasing from 86.0 to 171.1 ± 18.2 µS cm^−1^, while the EC values of CFA increased from 152.0 to 380.3 ± 42.1 µS cm^−1^. This can be explained by the increased dissolution of minerals due to lower pH values, suggesting improved bioavailability of these ions [35]. CFA is highly characterized by minerals and as such higher EC values were reported in the CFA in comparison to the soil samples. The EC of the substrate affects the transport of nutrients as well as the uptake of water by the plants. The TOC of all the samples ranged between 2.58–3.92%, which is an optimum amount supporting microorganisms, contributing to nutrient retention and improved structure of the substrate. The most desired TOC is actually in the range of 2–5% [36], because high TOC percentages can restrict the mobility of metals. The values of pH and EC are related to plant growth and therefore the improved values for the CFA samples suggest conditions that are beneficial to plant development.

### 3.2. Plant Growth in the Control Soil and CFA during the Pot Trials

After 14 weeks of the experiment, plant growth was measured and recorded, and an important growth was observed for the control and CFA plants, as depicted in Table 2. The control plant growth was increased by 18.9%, while the CFA plants reported 10.0% growth. These results indicate phytotoxicity, which can be attributed to the presence of pollutants such as toxic metals in the CFA and lower microbial activity that supports plant growth [24]. As it is seen in Table 2, the plants grown in the CFA were stunted as compared to those grown in the control soil.

### 3.3. Gas-Exchange Analysis

Gas-exchange measurements, including leaf photosynthetic rate (A), internal CO_2_ (Ci), stomatal conductance (Gs) and leaf transpiration (E), were recorded twice within the growing period. The measurements were performed on plants grown in four treatments, including controls and CFA plants assigned control soil and CFA-C_1_, CFA-C_2_ and CFA-C_3_, respectively. The values for A, which indicate CO_2_ assimilation, were higher for the controls, and slightly lower values were observed for the CFA plants, with lowest values found in CFA-C_3_ plants. This follows the same trend as plant growth, which was slightly lower for the CFA plants. The results revealed that there were no significant differences in the values of Gs, Ci and E for both the controls and the CFA plants. The WUE was calculated by dividing A by Gs and the results revealed that controls exhibit higher WUE in comparison to the CFA plants. Intercellular CO_2_ determines the flux of carbon dioxide into the leaf if the stomatal opening remains constant, as the closure of the stomata can lead to uncertainties. The results revealed no significant differences, which suggest that plants grown in CFA had accumulated significant biomass under harsh conditions. The concentration was found to be 259 ± 6.3 µmol (CO_2_) mol^−1^ air^−1^ for the CFA-C_1_ treatment, and a value of 258 ± 6.6 µmol (CO_2_) mol^−1^ air^−1^ was reported for the controls (Table 3). Plants grown in soil had higher WUE values than CFA plants, indicating that more water was necessary for the CFA plants, hence the watering schedule was slightly altered. The variation in photosynthetic characteristics can be due to the restricted movement of water, therefore restricting the supply of available of nutrients [32,37]. Optimum pH for nutrient bioavailability ranges between 6.0–6.5 and higher values affect their availability for root uptake, influencing the nutrient balance throughout the plant system [38].

### 3.4. Metal Analysis

ICP-MS was used to measure the concentrations of As, Cd, Co, Cr, Cu, Mn, Ni, Pb and Zn in CFA and control soil (before and after treatment) and roots and shoots of *H. splendidum* in order to assess their uptake, accumulation and translocation (Table 4 and Table 5). The baseline concentrations in the CFA and soil samples were determined in order to determine the removal efficiency of elements from the substrates (Table 4). The method validation was conducted by determining these elements in the CRM (SRM 1944, New York Waterway Sediments, National Institute of Standards and Technology, New York, NY, USA) and the results were within the certified values. The results revealed that CFA and the control soil media contained all the tested elements and their pattern was Mn > Cr > Zn > Ni > As > Cu > Co > Pb > Cd in CFA and Mn > Cr > Zn > Ni > Cu > Co > As > Pb > Cd in soil, as shown Table 4

The results obtained are above the permissible levels and suggest that the CFA and control soil samples are moderately contaminated with Cd [39,40]. The control soil medium had higher concentrations of other elements as compared to the CFA, except As and Cd. The amount of As and Cr in CFA were found to be 47.2 ± 4.1 and 131 ± 14.9 mg kg^−1^, respectively, which exceeded the permissible limit of Earth’s crust average concentrations (1.7 and 83 mg.kg^−1^ for As and Cr, respectively), indicating that CFA is polluted with these toxic metals [41].

Although Mn is essential for plant growth, a higher level can induce phytotoxicity in plants due to oxidative stress [42]. This study revealed that the CFA contained 456 ± 36.2 mg kg^−1^ Mn, and this is above the acceptable limit and therefore could contribute towards slow growth in CFA plants. However, *H. splendidum* recorded 15.8% removal efficiency of this element, indicating its tolerance toward metal toxicity. The concentrations of Pb were found to be moderate in the CFA (9.70 ± 1.3 mg kg^−1^), and the highest amount was found in the roots (5.60 ± 0.3 mg kg^−1^) as compared to the shoots (4.20 ± 0.06 mg kg^−1^). Similarly, a lower amount was found in the roots of *H. splendidum* for the controls, and this can suggest that mobility is restricted in the substrates with lower Pb amounts. The removal efficiency for Pb was 21.8%, indicating that Pb can be removed from CFA with the use of *H. splendidum*. High As levels for both the soil (63.0 ± 5.5 mg kg^−1^) and CFA (91.1 ± 7.7 mg kg^−1^) can be attributed to the combustion of coal [41]. According to the World Health Organization (WHO), the concentration of As in soil should be below 20 mg kg^−1^, indicating that the CFA and the soil are moderately polluted with As. Although unacceptable levels of metals were found in the CFA, all the elements were assimilated into the tissues of *H. splendidum*, recording between 15.8 and 56.7% removal efficiency as depicted in Table 5. The highest removal efficiencies were observed for As (48%), Cu (56.7%) and Zn (55.6%), suggesting that the plant is more effective in removing these elements and this is not related to the amount of the element in the original substrate.

This study revealed that *H. splendidum* can take up metals and assimilate them into its tissues (roots and shoots), as presented in Table 5. Most of the elements were accumulated in the roots, as compared to the shoots, for both the control soil and the CFA-grown plants. Higher concentrations of Cd, Co, Cd, Cu, Mn and Pb were found in the roots, while the shoots had higher amount of As, Cr and Zn for the soil-grown control plants, as shown in Table 5. In contrast, Cr was mostly accumulated in the roots of CFA-grown plants, while As and Zn were mostly accumulated in the shoots of CFA-grown plants. The concentration of Cr was found to be 69.8 ± 3.8 mg kg^−1^ in the shoots and 100 ± 7.7 mg kg^−1^ in the roots. The concentration of Ni was not significantly different between the shoots and the roots, reporting 45.9 ± 5.81 mg kg^−1^ in the shoots, while 42.4 ± 3.04 mg kg^−1^ was found in the roots.

Plants grown in soil accumulated and translocated higher concentrations of metals than the those grown in CFA. This can be explained by enough plant biomass in the control soil medium enhancing the accumulation and translocation of these metals in the roots and shoots, respectively. To enhance biomass accumulation, organic amendments can be applied to improve the CFA’s physicochemical properties, the microbial community and nutrient bioavailability, thus accelerating plant growth [43].

### 3.5. Assessing the Potential of H. splendidum for Phytoremediation of CFA-Polluted Sites

In this study, the phytoextraction and phytostabilization processes were performed on *H. splendidum* species planted in both control soil and CFA media. To estimate a phytoremediation potential of a plant, translocation factors (TFs) (see Equation (2), Section 2.6) and bioconcentration factors (BCFs) (see Equation (1), Section 2.6) are widely applied [25,27]. Metal uptake and bioaccumulation in plant species varied from metal to metal. Translocation of elements from the roots to the shoots is the measure used to evaluate phytoextraction, whereas in the phytostabilzation process, the metals are accumulated in the roots of the plant [44]. The BCF values of As, Cu and Zn in CFA and As and Zn in the control soil media were greater than 1, as depicted in Table 6. The remaining BCFs of Pb, Mn, Co, Ni, Cd, and Cr in the CFA medium, and the BCFs of Pb, Mn, Ni, Co, Cu and Cd in control soil medium, were less than 1. The TFs of Ni, Zn and As in CFA and As, Cr and Zn in the soil-grown control plants were greater than 1, while the TFs of Pb, Mn, Co, Cu, Cd and Cr in CFA-grown plants and the TFs of Cd, Co, Cu, Mn, Ni and Pb in the soil-grown control plants, were less than 1. However, all these metals with a TF-threshold value of < 1 were accumulated in the roots and not translocated to the leaves, except Ni, As and Zn in the CFA-grown plants and As, Cr and Zn in soil-grown control plants. Any metal with TF values > 1 shows that the plant has high efficiency to translocate those particular metals from the roots to the shoots, and a higher amount of metal in the root with <1 TF value shows an ability of the plant to harmonize between metal accumulation and translocation [45].

## 4. Conclusions

This study was able to highlight the potential of *H. splendidum* for phytoremediation of CFA-polluted sites in South Africa. The plants did not show high BCF and TFs values for all elements, but some of them, such as As, Cu and Zn, were greater than 1 in CFA-grown plants, which can make the plant species a good phytoextractor. This study showed an even distribution of metal contents in the soils and plant tissues, and it also showed low TFs for most elements, which also makes the plant species an attractive potential for the phytostabilization of trace metals. Although the CFA medium had restricted growth of some plants as compared to the control soils, the results showed an interesting removal efficiency ranging between 18.0 to 56.7%, with the highest values reported for Cu and Zn. From the obtained results, it can be concluded that *H. splendidum* has the potential to be used for plant-based remediation of CFA-polluted sites. The technique can be used to reduce the concentrations of trace metals from the CFA, and the CFA can become less toxic and more suitable for plantation. The observed gas-exchange parameters were very similar in both control soil and in the CFA substrates. Further studies of *H. splendidum* for different CFA treatments need to be conducted to enhance the plant’s phytoremediation capabilities. In summary, the metal concentrations in the CFA-grown plants were almost comparable with those in the soil-grown control plants, importantly the Cr, Zn and Mn concentrations were greater in the shoots for soil-grown control plants compared to the CFA-grown plants. Mn had a high concentration in the roots in both medium-grown plants. The results showed that *H. splendidum* was able to extract all metals from the substrates to its tissues, rendering it good a phytostabilizer and phytoextractor candidate for trace metals.

## Figures and Tables

**Table 1:** Chemical and physical properties of CFA and soil sampled before and after pot trials (*n* = 3).

Before Treatment	pH	EC (µScm^−1^)	TOC (%)
Control soil	5.72 ± 0.028 b	85 ± 0.57 b	3.35 ± 0.028 b
CFA	7.91 ± 0.020 a	152 ± 0.58 a	3.88 ± 0.020 a
F-statistics	38.45 ***	30.631 ***	230.52 ***
After planting			
Control soil	6.51 ± 0.06 b	171.1 ± 18.2 b	2.58 ± 0.05 b
CFA	7.47 ± 0.07 a	380.3 ± 42.11 a	3.47 ± 0.08 a
F-statistics	114.88 ***	20.789 *	78.93 ***

Note: ***: very highly significant (*p* < 0.001), *: significant (*p* < 0.05), EC: electrical conductivity, CFA: coal fly ash, TOC: total organic carbon, “a” values are higher than “b” values (a > b).

**Table 2:** Plant growth measurements recorded over 14 weeks (*n* = 3 ± SD).

Treatment	Plant Height (cm)-Week 1	Plant Height (cm)-Week 2	Plant Height (cm)-Week 6	Plant Height (cm)-Week 8	Plant Height (cm)-Week 11	Plant Height (cm)-Week 14	Increase (%)
Control soil	21.2 ± 1.2 a	22.1 ± 0.9 a	23.0 ± 1.1 a	23.9 ± 1.2 a	24.5 ± 1.4 a	25.2 ± 1.6 b	18.9
CFA	22.2 ± 0.9 a	22.6 ± 0.8 a	23.1 ± 1.0 a	23.4 ± 1.0 a	23.7 ± 1.0 a	24.4 ± 1.2 a	10.0
F-statistics	0.540 ns	0.723 ns	0.001 ns	0.119 ns	0.171 ns	0.158 ns	

Note: ns: non-significant. “a” values are higher than “b” values (a > b).

**Table 3:** Measured photosynthetic parameters of *Helichrysum splendidum*.

Treatment	A	Gs	Ci	E	WUE
	µmol (CO_2_) m^−2^ s^−1^	mol (H_2_O) m^−2^ s^−1^	µmol (CO_2_) mol^−1^ air^−1^	mol (H_2_O) m^−2^ s^−1^	µmol (CO_2_) m^−1^.H_2_O
Control soil	23.9 ± 1.1 a	0.08 ± 0.02 a	258.0 ± 6.6 a	1.96 ± 0.24 a	455.0 ± 56.0 a
CFA-C_1_	21.0 ± 0.8 b	0.07 ± 0.01 a	259.0 ± 6.3 a	2.25 ± 0.30 a	350.0 ± 24.0 b
CFA-C_2_	21.5 ± 0.9 b	0.07 ± 0.01 a	243.0 ± 14.9 a	2.11 ± 0.29 a	380.0 ± 32.0 a
CFA-C_3_	20.9 ± 0.8 c	0.07 ± 0.01 a	246.0 ± 6.8 a	2.27 ± 0.30 a	337.0 ± 46.0 b
F-statistics	2.57 *	0.32 ns	0.97 ns	0.397 ns	1.45 ns

Note: intercellular CO_2_ concentration (Ci), photosynthetic rate (A), transpiration efficiency (E), stomatal conductance (Gs) and water-use efficiency (WUE) of plants planted on five different soils. ns: non-significant, *: significant (*p* < 0.05). CFA-C_1_: coal fly ash treatment 1, CFA-C_2_: coal fly ash treatment 2 and CFA-C_3_: coal fly ash treatment 3. Values are means (*n* = 3) ± SD, statistically significant at *p* < 0.05 for A and non-significant at *p* > 0.05 for Gs, Ci, E and WUE. a means higher than b, b means higher than c (a > b > c).

**Table 4:** Baseline measurements of CFA and control soil samples before treatment.

Metal	Control Soil Baseline (mg kg^−1^)	CFA Baseline (mg kg^−1^)
As	63.0 ± 5.5	91.1 ± 7.7
Cd	4.4 ± 0.8	6.5 ± 2.2
Co	36.4 ± 4.4	18.6 ± 3.9
Cr	309.0 ± 28.3	185.0 ± 17.0
Cu	59.2 ± 4.2	23.5 ± 2.2
Mn	1060.0 ± 60.0	456.0 ± 36.0
Ni	96.5 ± 7.8	87.2 ± 7.4
Pb	21.7 ± 1.3	12.4 ± 1.0
Zn	224.0 ± 19.2	177.0 ± 15.3

Note: Values are means (*n* = 3) ± SD, statistically significant at *p* < 0.05.

**Table 5:** Analysis of heavy metals in CFA, soil and plant samples (mg kg^−1^) after the 14-week pot trials.

Metal	Control Medium	Control Shoots	Control Roots	CFA Medium	CFA Shoots	CFA Roots	%Removal (CFA)
As	28.6 ± 1.5	52.3 ± 3.1	49.2 ± 2.2	47.2 ± 4.1	62.7 ± 2.7	57.9 ± 6.2	48.2
Cd	2.4 ± 0.0	1.5 ± 0.1	1.9 ± 0.1	4.5 ± 0.3	2.2 ± 0.8	2.7 ± 0.1	30.8
Co	28.8 ± 1.5	7.6 ± 0.1	16.3 ± 1.9	10.6 ± 0.6	3.2 ± 0.4	5.9 ± 0.1	18.0
Cr	178 ± 15.2	132 ± 1.1	111 ± 5.1	131 ± 14.9	69.8 ± 3.7	100 ± 7.7	29.1
Cu	50.6 ± 4.3	39.0 ± 2.1	40.2 ± 3.1	30.8 ± 4.0	30.8 ± 1.2	36.5 ± 2.1	56.7
Mn	910 ± 35.1	375 ± 27.3	697 ± 35.0	384 ± 23.1	178 ± 14.0	259 ± 14.0	15.8
Ni	81.1 ± 7.7	36.0 ± 3.1	44.1 ± 5.1	52.6 ± 6.9	45.9 ± 5.8	42.4 ± 3.0	39.7
Pb	16.1 ± 2.1	5.0 ± 0.2	7.1 ± 0.3	9.7 ± 1.2	4.2 ± 0.0	5.6 ± 0.3	21.8
Zn	130 ± 6.5	150 ± 13.4	131 ± 11.1	76.4 ± 6.3	86.4 ± 7.3	78.6 ± 8.2	55.6

Note: Values are means (*n* = 3) ± SD, statistically significant at *p* < 0.05.

**Table 6:** Bioconcentration factors and translocation factors of metals in CFA and soil.

Metals	BCF-Control Soil	TF-Control Soil	BCF-CFA	TF-CFA
As	1.70	1.06	1.22	1.08
Cd	0.79	0.79	0.50	0.81
Co	0.56	0.46	0.55	0.54
Cr	0.62	1.19	0.76	0.76
Cu	0.79	0.97	1.19	0.84
Mn	0.76	0.53	0.67	0.68
Ni	0.54	0.81	0.80	1.08
Pb	0.44	0.70	0.57	0.75
Zn	1.02	1.14	1.03	1.12

Note: BCF-control soil: bioconcentration factor of control soil, TF-control soil: translocation factor of control soil, BCF-CFA: bioconcentration factor of coal fly ash, and TF-CFA: translocation factor of coal fly ash.

## Data Availability

Data are contained within the article.

## References

[1] Heidrich C., Feuerborn H.J., Weir A. Coal Combustion Products: A Global Perspective. Proceedings of the World of Coal Ash Conference.

[2] Yadav V.K., Gacem A., Choudhary N., Rai A., Kumar P., Yadav K.K., Islam S. (2022). Status of coal-based thermal power plants, coal fly ash production, utilization in India and their emerging applications. Minerals.

[3] Dwivedi A., Jain M.K. (2014). Fly ash-waste management and overview: A Review. Recent Res. Sci. Technol..

[4] Bhatt A., Priyadarshini S., Mohanakrishnan A.A., Abri A., Sattler M., Techapaphawit S. (2019). Physical, chemical, and geotechnical properties of coal fly ash: A global review. Case Stud. Constr. Mater..

[5] Kelechi S.E., Adamu M., Uche O.A.U., Okokpujie I.P., Ibrahim Y.E., Obianyo I.I. (2022). A comprehensive review on coal fly ash and its application in the construction industry. Cogent Eng..

[6] Van der Merwe E.M., Prinsloo L.C., Mathebula C.L., Swart H.C., Coetsee E., Doucet F.J. (2014). Surface and bulk characterization of an ultrafine South African coal fly ash with reference to polymer applications. Appl. Surf. Sci..

[7] Vilakazi A.Q., Ndlovu S., Chipise L., Shemi A. (2022). The recycling of coal fly ash: A review on sustainable developments and economic considerations. Sustainability.

[8] Ivanova T.S., Panov Z., Blazev K., Paneva V.Z. (2011). Investigation of Fly Ash Heavy Metals Content and Physico-Chemical Properties from Thermal Power Plant, Republic of Macedonia. Int. J. Eng. Sci. Technol..

[9] Rashidi N.A., Yusup S. (2016). Overview on the potential of coal-based bottom ash as low-cost adsorbents. ACS Sustain. Chem. Eng..

[10] Gajić G., Djurdjević L., Kostić O., Jarić S., Mitrović M., Pavlović P. (2018). Ecological Potential of Plants for Phytoremediation and Ecorestoration of Fly Ash Deposits and Mine Wastes. Front. Environ. Sci..

[11] Jambhulkar H.P., Shaikh S.M.S., Kumar M.S. (2018). Fly ash toxicity, emerging issues and possible implications for its exploitation in agriculture; Indian scenario. Chemosphere.

[12] Burgess R.M., Perron M.M., Friedman C.L., Suuberg E.M., Pennell K.G., Cantwell M.G., Ryba S.A. (2009). Evaluation of the effects of coal fly ash amendments on the toxicity of a contaminated marine sediment. Environ. Toxicol. Chem. Int. J..

[13] Qadir M., Schubert S., Oster J.D., Sposito G., Minhas P.S., Cheraghi S.A., Saqib M. (2018). High-magnesium waters and soils: Emerging environmental and food security constraints. Sci. Total Environ..

[14] Ahmadpour P., Ahmadpour F., Maumud T.M.M., Arifin Abdu Soleimani M., Hosseini Tayefe F. (2012). Phytoremediation of heavy metals: A green technology. Afr. J. Biotechnol..

[15] Jadia C.D., Fulekar M.H. (2009). Phytoremediation of Heavy Metals: Recent Techniques. Afr. J. Biotechnol..

[16] Banda M.F., Makgalaka N.S., Combrinck S., Regnier T. (2021). Five-weeks pot trial evaluation of phytoremediation potential of *Helichrysum splendidum* Less. For copper- and lead-contaminated soils. Int. J. Environ. Sci. Technol..

[17] Zgorelec Z., Bilandzija N., Knez K., Galic M., Zuzul S. (2020). Cadmium and Mercury Phytostabilization from Soil Using *Miscanthus giganteus*. Nature.

[18] Li X., Huang L. (2015). Toward a new paradigm for tailings phytostabilization-nature of the substrates, amendment options, and anthropogenic pedogenesis. Crit. Rev. Environ. Sci. Technol..

[19] Ghosh M., Singh S.P. (2005). A Review on Phytoremediation of Heavy Metals and Utilization of Its By-Products. Asian J. Energy Environ..

[20] Nwoko C.O. (2010). Trends in Phytoremediation of Toxic Elemental and Organic Pollutants. Afr. J. Biotechnol..

[21] Eltaher G.T., Ahmed D.A., El-Beheiry M., El-Din A.S. (2019). Biomass estimation and heavy metal accumulation by *Pluchea dioscoridis* (L.) DC. in the Middle Nile Delta, (Egypt): Perspectives for phytoremediation. S. Afr. J. Bot..

[22] Bakshe P., Jugade R. (2023). Phytostabilization and rhizofiltration of toxic heavy metals by heavy metal accumulator plants for sustainable management of contaminated industrial sites: A comprehensive review. J. Hazard. Mater. Adv..

[23] Ali H., Khan E., Sajad M.A. (2013). Phytoremediation of heavy metals-Concepts and applications. Chemosph. Environ. Chem..

[24] Chen Y., Fan Y., Huang Y., Liao X., Xu W., Zhang T. (2024). A comprehensive review of toxicity of coal fly ash and its leachate in the ecosystem. Ecotoxicol. Environ. Saf..

[25] Maiti S.K., Ghosh D., Raj D. (2022). Phytoremediation of fly ash: Bioaccumulation and translocation of metals in natural colonizing vegetation on fly ash lagoons. Handbook of Fly Ash.

[26] Zhang W., Cai Y., Tu C., Ma L.Q. (2002). Arsenic speciation and distribution in an arsenic hyperaccumulating plant. Sci. Total Environ..

[27] Yoon J., Cao X., Zhou Q., Ma L.Q. (2006). Accumulation of Pb, Cu, and Zn in native plants growing on a contaminated Florida site. Sci. Total Environ..

[28] Hosman M.E., El-Feky S.S., Elshahawy M.I., Shaker E.M. (2017). Mechanism of phytoremediation potential of flax (*Linum usitatissimum L.)* to Pb, Cd and Zn. Asian J. Plant Sci. Res..

[29] Brunetti G., Ruta C., Traversa A., D’Ambruoso G., Tarraf W., De Mastro F., DeMastro G., Cocozza C. (2018). Remediation of a heavy metals contaminated soil using mycorrhized and non-mycorrhized *Helichrysum italicum* (Roth) Don. Land Degrad. Dev..

[30] Fernández S., Poschenrieder C., Marcenò C., Gallego J.R., Jiménez-Gámez D., Bueno A., Afif E. (2017). Phytoremediation capability of native plant species living on Pb-Zn and Hg-As mining wastes in the Cantabrian range, north of Spain. J. Geochem. Explor..

[31] Schumacher B.A. (2002). Methods for the Determination of Total Organic Carbon (TOC) in Soils and Sediments.

[32] Makoi J.H., Chimphango S.B., Dakora F.D. (2010). Photosynthesis, water-use efficiency and δ^13^C of five cowpea genotypes grown in mixed culture and at different densities with sorghum. Photosynthetica.

[33] Siyar R., Doulati Ardejani F., Norouzi P., Maghsoudy S., Yavarzadeh M., Taherdangkoo R., Butscher C. (2022). Phytoremediation potential of native hyperaccumulator plants growing on heavy metal-contaminated soil of Khatunabad copper smelter and refinery, Iran. Water.

[34] Ma W., Tang S., Dengzeng Z., Zhang D., Zhang T., Ma X. (2022). Root exudates contribute to belowground ecosystem hotspots: A review. Front. Microbiol..

[35] Roques S., Kendall S., Smith K., Price P.N., Berry P. (2013). A Review of the Non-NPKS Nutrient Requirements of UK Cereals and Oilseed Rape.

[36] Cheng S., Gao X., Cao L., Wang Q., Qiao Y. (2020). Quantification of Total Organic Carbon in Ashes from Smoldering Combustion of Sewage Sludge via a Thermal Treatment-TGA Method. Am. Chem. Soc..

[37] Hatfield J.L., Dold C. (2019). Water-use efficiency: Advances and challenges in a changing climate. Front. Plant Sci..

[38] Ferrarezi R.S., Lin X., Gonzalez Neira A.C., Tabay Zambon F., Hu H., Wang X., Huang J.H., Fan G. (2022). Substrate pH influences the nutrient absorption and rhizosphere microbiome of Huanglongbing-affected grapefruit plants. Front. Plant Sci..

[39] Asgher M., Khan M.I.R., Anjum N.A., Khan N.A. (2015). Minimising toxicity of cadmium in plants—Role of plant growth regulators. Protoplasma.

[40] Zulfiqar U., Jiang W., Xiukang W., Hussain S., Ahmad M., Maqsood M.F., Mustafa A. (2022). Cadmium phytotoxicity, tolerance, and advanced remediation approaches in agricultural soils; a comprehensive review. Front. Plant Sci..

[41] Altıkulaç A., Turhan S., Kurnaz A., Gören E., Duran C., Hançerlioğulları A., Uğur F.A. (2022). Assessment of the enrichment of heavy metals in coal and its combustion residues. ACS Omega.

[42] Khoshru B., Mitra D., Nosratabad A.F., Reyhanitabar A., Mandal L., Farda B., Mohapatra P.K.D. (2023). Enhancing manganese availability for plants through microbial potential: A sustainable approach for improving soil health and food security. Bacteria.

[43] Pandey V.C., Singh J.S., Singh R.P., Singh N., Yunus M. (2011). Arsenic hazards in coal fly ash and its fate in Indian scenario. Resour. Conserv. Recycl..

[44] Chowdhury A., Maiti S.K. (2016). Assessing the ecological health risk in a conserved mangrove ecosystem due to heavy metal pollution: A case study from Sundarbans Biosphere Reserve, India. Hum. Ecol. Risk Assess. Int. J..

[45] Iya N.I.D., Assim Z.B., Ipor I.B., Omolayo A.O., Umaru I.J., Jume B.H. (2018). Accumulation and translocation of heavy metals by Acalypha wilkesiana parts in the phytoextraction of contaminated soil. Indones. J. Chem..

